# The Startle Disease Mutation E103K Impairs Activation of Human Homomeric α1 Glycine Receptors by Disrupting an Intersubunit Salt Bridge across the Agonist Binding Site*

**DOI:** 10.1074/jbc.M116.767616

**Published:** 2017-02-07

**Authors:** Fatemah Safar, Elliot Hurdiss, Marios Erotocritou, Timo Greiner, Remigijus Lape, Mark W. Irvine, Guangyu Fang, David Jane, Rilei Yu, Marc A. Dämgen, Philip C. Biggin, Lucia G. Sivilotti

**Affiliations:** From the ‡ Department of Neuroscience, Physiology and Pharmacology, University College London, Gower Street, London WC1E 6BT, United Kingdom,; the ¶ Department of Biochemistry, Structural Bioinformatics and Computational Biochemistry, University of Oxford, South Parks Road, Oxford OX1 3QU, United Kingdom,; the § School of Physiology and Pharmacology, University of Bristol, University Walk, Bristol BS8 1TD, United Kingdom, and; the ‖ Key Laboratory of Marine Drugs, Chinese Ministry of Education, School of Medicine and Pharmacy, Ocean University of China, Qingdao 266003, China

**Keywords:** glycine receptor, homology modeling, ion channel, mutagenesis, patch clamp, site-directed mutagenesis, agonists, efficacy, potency

## Abstract

Glycine receptors (GlyR) belong to the pentameric ligand-gated ion channel (pLGIC) superfamily and mediate fast inhibitory transmission in the vertebrate CNS. Disruption of glycinergic transmission by inherited mutations produces startle disease in man. Many startle mutations are in GlyRs and provide useful clues to the function of the channel domains. E103K is one of few startle mutations found in the extracellular agonist binding site of the channel, in loop A of the principal side of the subunit interface. Homology modeling shows that the side chain of Glu-103 is close to that of Arg-131, in loop E of the complementary side of the binding site, and may form a salt bridge at the back of the binding site, constraining its size. We investigated this hypothesis in recombinant human α1 GlyR by site-directed mutagenesis and functional measurements of agonist efficacy and potency by whole cell patch clamp and single channel recording. Despite its position near the binding site, E103K causes hyperekplexia by impairing the efficacy of glycine, its ability to gate the channel once bound, which is very high in wild type GlyR. Mutating Glu-103 and Arg-131 caused various degrees of loss-of-function in the action of glycine, whereas mutations in Arg-131 enhanced the efficacy of the slightly bigger partial agonist sarcosine (*N*-methylglycine). The effects of the single charge-swapping mutations of these two residues were largely rescued in the double mutant, supporting the possibility that they interact via a salt bridge that normally constrains the efficacy of larger agonist molecules.

## Introduction

Ion channels that belong to the pentameric ligand-gated superfamily (pLGIC)^5^ are found both in prokaryotic and eukaryotic organisms. In vertebrates, including man, pLGIC mediate fast synaptic transmission both in the periphery (see for instance, the cation permeable, nicotinic ACh receptors at the neuromuscular junction) and in the central nervous system (*cf*. the inhibitory, anion-permeable GABA and glycine receptors). pLGIC are activated by the binding of agonist/neurotransmitters to canonical binding sites at the extracellular interface of two subunits. Each receptor type recognizes as agonists a fairly narrow range of ligand molecules. However, in the superfamily as a whole, the group of chemical structures that can activate these channels is remarkably diverse, from small amines such as ACh or propylamine, to amino acids (glycine, GABA, and glutamate), to aromatic compounds (histamine, serotonin, tyramine, and nicotine). How pLGIC achieve this level of agonist recognition is still an area of intense investigation (1).

Structural data from several pLGIC members show that channel subunits have a conserved fold and that the main structural features of the agonist binding site, as first hypothesized in the early 1990s (2), are shared across the superfamily. Thus, the site is made by “loops” A, B, and C from the outer β sheet of the anticlockwise (principal) subunit and by loops D to G from the inner β sheet of the clockwise (complementary) subunit. The loops contain many aromatic residues, an “aromatic box” (3, 4) whose side chains form cation-π interactions with the positively charged moiety of pLGIC agonists. The actual aromatic residues that make the “box” differ across pLGIC subtypes, and probably so does the size of the aromatic box (5).

Combining structural and functional data with homology modeling suggests that few of the loop residues form direct bonds with the agonist, whereas others contribute to holding these key residues in place (1, 6, 7). Which agonists are recognized and what sets their efficacy is likely to be influenced by other properties of the binding site, such as its size and flexibility both at rest and after activation, but we do not know how these are determined in the protein.

For pLGICs as for other channels, important clues about the function of the different structural domains have come from the discovery and characterization of channelopathy mutations that cause inherited disease in man. The human disease associated with impaired glycinergic transmission is called startle disease or hyperekplexia, and is caused most often by loss-of-function mutations of glycine receptors (GlyR), particularly in the α1 subunit (8). Startle disease mutations are thought to exert their effects largely by damaging receptor gating, namely how well the GlyR opens once it has bound the neurotransmitter glycine (9, 10). This hypothesis is largely based on the analysis of the first mutations to be characterized, such as K276E and Q266H (9–12). These residues are in the transmembrane domain of the channel, where the channel gate is and where most startle mutations are found. Relatively few startle mutations are in the receptor binding site and little is known of how they may act. If these disrupt binding, anything short of a profound disruption of agonist binding is unlikely to damage glycinergic synaptic transmission enough to produce a startle phenotype, because glycine in the synaptic cleft reaches high concentrations of 2.2–3.5 mm (13) and this should provide a safety margin. A recent report (14) has shown that a startle mutation lethal in the mouse, N46K α1 GlyR (near the principal side of the binding site) may exert its effects by speeding up GlyR deactivation. Startle mutations that are in the binding site and do not abolish agonist binding may be particularly interesting to investigate, as they are likely to act by interfering with the transduction of the agonist binding signal toward the pore. This “gating aspect” of the binding site function is poorly understood, but important, as it must contribute to how agonist efficacy is determined.

**FIGURE 1.**
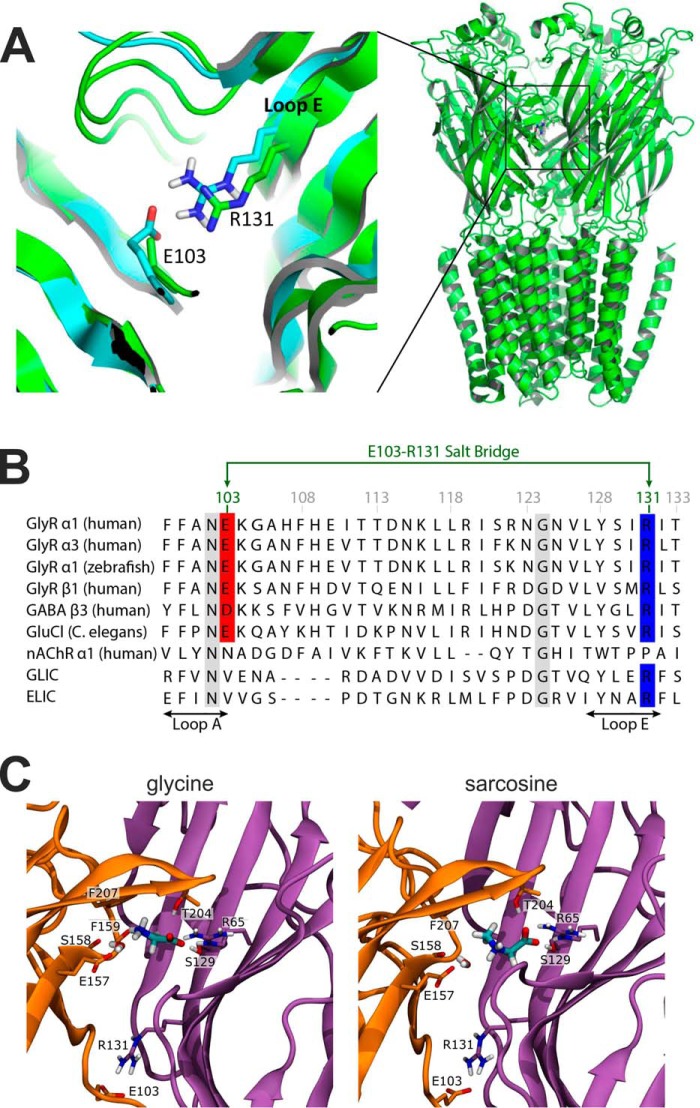
**Homology modeling reveals a salt-bridge between residues Glu-103 and Arg-131.** *A,* location of the Glu^103^-Arg^131^ salt-bridge as predicted by a GlyR homology model (*cyan*)(7) built using GluCl as template (PDB code 2XYS) (16). The model is overlaid with the recent cryo-EM structure (*green*) of the zebrafish α1 GlyR (PDB 3JAE) (17). Note that the Glu-103 side chain is not fully resolved in the cryo-EM structure, but is certainly within distance to form the salt bridge as postulated by the model. *B,* an amino acid alignment of this region for nine members of the pLGIC superfamily showing the position of the salt bridge between Glu-103 (*red*) and Arg-131 (*blue*) and its conservation in glycine receptors. Other conserved positions are indicated by a *gray* background. *C,* the agonist binding mode and the Glu^103^-Arg^131^ salt bridge in a GlyR homology model based on the zebrafish α1 GlyR structure (PDB 3JAE) (17). Glycine (*left*) and sarcosine (*right*) are shown in the final frame after 500 ns simulation time. *Orange*, principal subunit; *purple*, complementary subunit. For both ligands, the carboxyl group is sandwiched by hydrogen bonds with Thr-204 and Ser-129 and is further stabilized by a salt bridge with Arg-65. The ammonium moiety interacts with a water molecule in the binding pocket, and the water is stabilized by hydrogen bonds to Glu-157 and to the backbone carbonyl oxygen of Ser-158 (as in Ref. 7). The ammonium group of glycine engages in an additional cation-π interaction with Phe-207 and forms a hydrogen bond with the backbone carbonyl group of Phe-159. This is sterically hindered for sarcosine by its additional *N*-methyl group.

The startle mutation E103K in loop A, on the principal side of the binding pocket (Fig. 1, *A* and *B*), was first described in a patient whose second α1 allele carried a frameshift mutation (15). Our homology model of the GlyR (7) based on the structure of GluCl (16) suggested that the side chain of Glu-103 is likely to form a salt bridge across the binding site with Arg-131 in loop E of the complementary subunit. To our knowledge, no hyperekplexia mutations have been reported for Arg-131. Molecular dynamics simulations on a new model built on the recent cryo-EM structure of the zebrafish α1 GlyR (17) confirm that the salt bridge is present and show that it is conformationally stable (at least on a 500-ns time scale). In the present study we confirmed the interaction between these two residues and found that, despite its binding site location, the hyperekplexia E103K mutation affected only glycine efficacy. The effect of mutating Glu-103 and Arg-131 was different for the full agonist glycine and the bulkier, partial agonist sarcosine.

## Results

### 

#### 

##### The Hyperekplexia Mutation E103K Decreases Both the Potency and the Efficacy of Glycine on α1 GlyR

Fig. 1*A* shows the position of the Glu-103 residue in a view taken from the GlyR homology model (7) that we produced (cyan) from the structure of GluCl (34% sequence identity with the α1 GlyR (16). Glu-103 is located at (or near) loop A of the principal (+) subunit (on the *left* in Fig. 1*A*, *orange* in Fig. 1*C*), and its negatively charged side chain is near the positively charged side chain of Arg-131 in loop E on the complementary (−) side of the binding site (on the *right* in Fig. 1A, *purple* in Fig. 1*C*). In the model, distances between side chain hydrogen atoms of Arg-131 to the side chain oxygen atoms of Glu-103 are 2.5∼2.8 Å, a range compatible with the presence of a salt bridge, which conceivably might have a stabilizing effect both on loops A and E and tighten the size of the binding site. The homology model is very similar to the structure of the zebrafish α1 GlyR (*green* in Fig. 1*A*; Protein Data Bank (PDB) code 3JAE) (17), where the position of the side chains of the two amino acids is not well defined. To investigate this further we performed molecular dynamics simulations (500 ns each for glycine and sarcosine bound) on a new model of human α1 GlyR based upon the zebrafish α1 GlyR structure (with glycine bound; PDB code 3JAE) (17); the domains in the structure have 94% sequence identity with the human α1 GlyR). In both cases the salt bridge was conformationally stable and present for at least 90% of the simulation time (90% for glycine bound and 97% for sarcosine) as averaged for all five binding pockets. The binding modes and salt bridge behavior were similar for glycine and sarcosine (Fig. 1*C*, *left* and *right*).

Glu-103 and Arg-131 are at the back of the binding site, toward the extracellular vestibule of the pore. Glu-103 is adjacent to residue Lys-104, whose charged side chain is likely to be exposed to the pore vestibule, and is one of the conductance determinants in pLGIC channels (18, 19). In our model, the charged side chains of both Glu-103 and Arg-131 are far from the center of the pore, at a distance of ∼16 Å, and are not exposed to the interior of the pore. Thus, it is unlikely that Glu-103 and Arg-131 affect conductance directly, but we cannot exclude that mutating either residue may change conductance by perturbing the position of nearby residue Lys-104.

Interestingly, both the GluCl and the zebrafish homology models show that Glu-103 and Arg-131 residues are also at some distance from the agonist (∼8 Å from glycine for example; data not shown), and thus any direct interaction with the agonist is expected to be minor as well. Any effects are therefore likely to be indirect, and to be exerted by affecting other residues, which do form direct bonds with glycine, such as loop A Phe-99 or loop E Ser-129. The salt bridge between Glu-103 and Arg-131 might be needed to maintain the correct conformation of loop A, which in turn influences the direct bonds of other residues with the agonist. An alignment of the amino acid sequences in loops A and E of nine pLGIC channels is shown in Fig. 1*B*. The Glu/Arg pair of residues in these locations are conserved in a subset of the inhibitory pLGIC receptors, namely all the GlyR subunits (α1–4 and β), irrespective of species, in *Caenorhabditis elegans* GluCl and in a few GABA receptor subunits, but is absent in the cationic channels (nicotinic, 5-HT_3_, GLIC, and ELIC).

In light of our considerations from the homology models, and our hypothesis that a salt bridge between Glu-103 and Arg-131 stabilizes loops A and E and tightens the binding site, we decided to test the effects of the E103K startle mutation on the GlyR responses to the full agonist glycine and to the partial agonist sarcosine (20). Sarcosine (*N*-methylglycine) is slightly bulkier than glycine (see the structures of the two agonists in Fig. 1*C*) because of the methyl group substitution on the amino group, which is likely to be oriented toward the back of the binding site (7).

**FIGURE 2.**
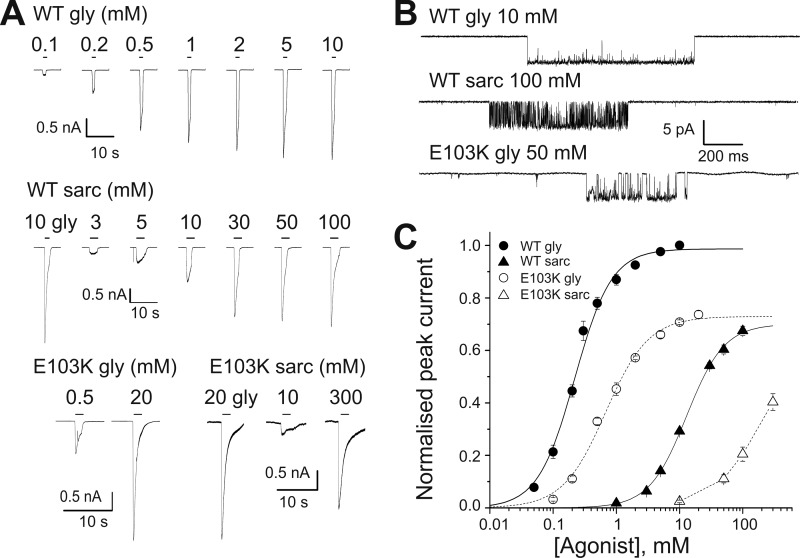
**The E103K startle mutation reduces the sensitivity of α1 GlyR to both glycine and sarcosine and impairs channel gating.** *A,* representative whole-cell current responses evoked by U-tube agonist application to HEK 293 cells expressing WT α1 GlyR (*upper panels*, 2 different cells) or E103K α1 GlyR (*lower panel*, 2 different cells). *Black bars above* the traces show the timing of the applications. Panels also show the responses to a saturating concentration of glycine obtained in the cells used for the sarcosine concentration-response curves. *B,* clusters of single channel activity elicited by saturating concentrations of glycine (*upper panel*) or sarcosine (*middle panel*) on WT α1 GlyR or (glycine only) on E103K GlyR (*lower panel*; cell-attached configuration, channel openings downwards). Note the decreased single channel *P*_open_ in mutant receptors. *C*, whole cell concentration-response curves to glycine and sarcosine in WT (*n* = 7 and 10, respectively) and E103K α1 GlyR (*n* = 4 and 3, respectively). *Curves* are scaled to the maximum single channel *P*_open_ measured at saturating glycine or sarcosine; for sarcosine on E103K GlyR, we could not measure the maximum single channel *P*_open_ and the curve is scaled indirectly (*e.g.* responses to sarcosine are scaled to the macroscopic maximum response to glycine in the same cell, and then scaled to the maximum glycine single channel *P*_open_).

We began our investigation by obtaining whole cell concentration-response curves from HEK 293 cells expressing homomeric GlyR. Fig. 2*A* shows typical currents responses for the wild type α1 GlyR (*top two panels*) and the E103K mutant (*bottom panel*) to the agonists glycine and sarcosine. Glycine is a full agonist on homomeric α1 GlyR (21), but the traces in Fig. 2*A* (*middle panel*) show that sarcosine is a partial agonist, and its maximum current responses reach only 80% of those to glycine when the agonists are applied to the same cell at saturating concentrations (10 mm for glycine and 100 mm for sarcosine; Table 1). The time course of the currents was similar for both agonists, and desensitization became apparent at EC_30_-EC_50_ for both glycine and sarcosine on WT and mutant receptors.

**TABLE 1:** **Properties of wild type and mutant human recombinant α1 GlyR in patch clamp experiments**

	Whole cell dose-response curves					Single channel measurements			
	EC_50_	*I*_max_	*I*_max_*/I*_GLY max_	*n*_H_	*n*	Concentration	max*P*_open_ (# clusters)	Amplitude	*n*
	*mm*	*nA*				*mm*		*pA*	
**Glycine**									
WT	0.23 ± 0.02	8 ± 2		1.58 ± 0.07	10	10	0.987 ± 0.002 (49)	5.2 ± 0.2	9
E103K	0.71 ± 0.11	2.7 ± 1.4		1.32 ± 0.06	3	50	0.73 ± 0.07 (13)	5.2 ± 0.1	3
E103A	0.43 ± 0.05	5.9 ± 0.3		1.24 ± 0.06	3	100	0.97 ± 0.01 (11)	5.6 ± 0.2	3
E103R	1.4 ± 0.4	12.8 ± 1.6		0.78 ± 0.03	8	200	0.87 ± 0.02 (11)	5.8 ± 0.1	3
R131A	0.19 ± 0.03	8.7 ± 1.9		0.90 ± 0.01	5	30	0.95 ± 0.01 (16)	4.58 ± 0.04	2
R131E	1.6 ± 0.2	1.5 ± 0.3		1.6 ± 0.1	4	50	0.93 ± 0.02 (28)	3.90 ± 0.03	2
R131E/E103R	0.43 ± 0.04	3.7 ± 0.4		0.95 ± 0.02	5	100	0.996 ± 0.001 (9)	4.8 ± 0.1	5

**Sarcosine**									
WT	14 ± 1	5.1 ± 0.6	0.80 ± 0.03	1.51 ± 0.04	7	100	0.70 ± 0.03 (22)	5.6 ± 0.3	4
E103K	>80	1.5 ± 0.4	0.62 ± 0.04	1.37 ± 0.03	4	300	Very heterogeneous	4.2 ± 0.1	8
E103A	23 ± 3	5.1 ± 1.7	0.76 ± 0.03	1.8 ± 0.2	4	100	0.67 ± 0.08 (8)	4.5 ± 0.4	4
E103R	17 ± 3	2.6 ± 0.8	0.84 ± 0.09	1.22 ± 0.05	5	200	0.79 ± 0.03 (31)	5.0 ± 0.1	4
R131A	4.5 ± 0.5	6.0 ± 1.1	0.93 ± 0.02	1.4 ± 0.1	5	100	0.95 ± 0.01 (37)	5.4 ± 0.1	3
R131E	75 ± 2	6.6 ± 0.9	0.81 ± 0.03	2.05 ± 0.03	6	300	0.91 ± 0.04 (21)	4.7 ± 0.1	2
R131E/E103R	15 ± 2	8.1 ± 2.3	0.95 ± 0.04	1.28 ± 0.07	8	100	0.97 ± 0.01 (23)	4.9 ± 0.3	8

The E103K mutation reduced the channel sensitivity to agonists. The effect was relatively small for glycine, whose EC_50_ increased from 0.23 ± 0.02 to 0.71 ± 0.11 mm (*n* = 10 and 3, respectively; Fig. 2*A*, *filled* and *hollow circles* in Fig. 2*C*; Table 1). The loss of potency of sarcosine was much greater. In the mutant this prevented us from obtaining a full sarcosine dose-response curve (because of the very high concentrations required to saturate the curve). Thus we could not establish whether the mutation changed the sarcosine maximum response relative to glycine. Such a decrease in the relative maximum response to a partial agonist would have been the simplest way to check whether this binding site mutation impairs channel gating.

An even better way to assess whether a mutation has an effect on gating is to measure the channel maximum open probability in single channel records. The measurement of cluster open probability has the advantage (*versus* whole cell macroscopic responses curves) of measuring an absolute open probability value that is not affected by the level of expression of the different channels examined or by changes in the channel conductance produced by the mutations. In addition, cluster open probability measures only changes in receptor activation and is not affected by desensitization (as desensitized intervals are not included in the analysis). Because of that, in Fig. 2*C* (and the following figures) we display concentration-response curves as whole cell responses *scaled* to the maximum open probability measured by single channel analysis.

The traces in Fig. 2*B* show clusters of single channel activity in cell-attached patches at saturating agonist concentrations. The glycine traces (*top*) show that the channel exposed to 10 mm glycine is practically either desensitized or open all the time, with a very high maximum *P*_open_ of 0.987 ± 0.002 (*n* = 49 clusters from 9 patches; measured as cluster open time/total cluster time), confirming that glycine is a very efficacious agonist on WT homomeric GlyR (21). In agreement with the whole cell data, single-channel WT clusters activated by a saturating sarcosine concentration (100 mm; Fig. 2*B*, *middle trace*) have a lower maximum *P*_open_ of 0.70 ± 0.03 (*n* = 22) and therefore confirm that sarcosine is a partial agonist in WT GlyR. The *bottom trace* of Fig. 2*B* shows channel activity evoked by glycine in the E103K mutant. These openings show that the mutation did not affect conductance, as their amplitude is similar to that of WT channels (*right-hand column* in Table 1). Mutating the adjacent residue Lys-104 to glutamate produces a 22% conductance decrease in the α1 homomeric GlyR, but Lys-104 is likely to be exposed to the channel pore (18). Mutant channel activity showed distinct clustering, with an open probability of 0.73 ± 0.07 (*n* = 13) to saturating glycine. Single-channel activity of E103K mutants in response to 300 mm sarcosine was not informative, not only because we do not know whether this concentration produces maximum activation, but also because clusters were sometimes hard to define unambiguously and because there was substantial heterogeneity in the channel open probability from cluster to cluster (data not shown).

Thus single channel data confirm that the E103K mutation must impair channel gating elicited by the full agonist glycine. Many mutations that produce loss of gating function have similar effects on all agonists, but given the position of this mutation is near the binding site, we investigated this further.

##### The Effect of Mutating Residues Glu-103 and Arg-131 on the Agonist Action of Glycine and Sarcosine Is Different

To test the hypothesis that there is a salt bridge between Glu-103 and Arg-131, we mutated both residues individually to an alanine and also reversed the side chain charges, to arginine and glutamate, respectively.

**FIGURE 3.**
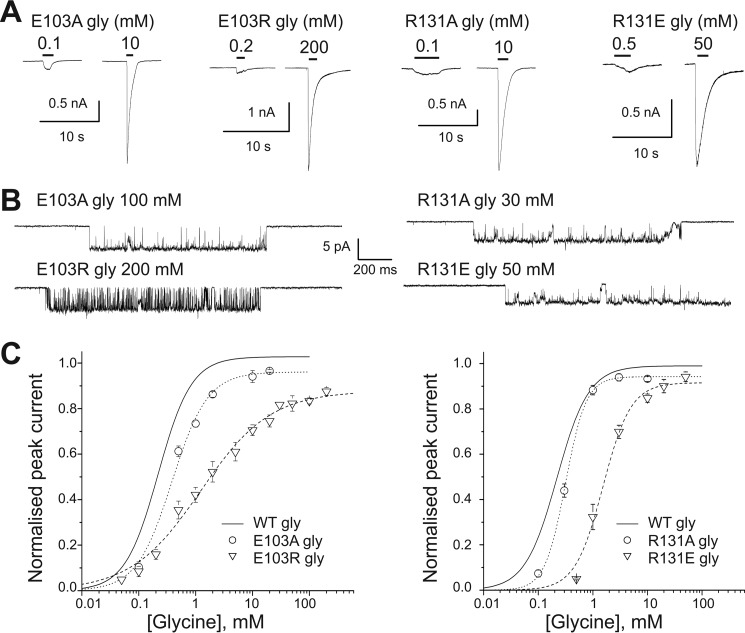
**Reversing the side chain charge in positions Glu-103 or Arg-131 changes the potency and efficacy of glycine.** *A,* representative whole cell current responses to glycine applied to GlyR α1 mutants. *B,* clusters of single-channel mutant GlyR activity elicited in cell-attached patches by saturating concentrations of glycine. *C,* whole cell concentration-response curves for the effect of glycine on E103A, E103R, R131A, and R131E mutant GlyR (*n* = 3, 8, 5, and 4, respectively). *Curves* are scaled to the maximum *P*_open_ obtained from single channel recordings. The WT concentration-response curve for glycine is shown for comparison (*solid line*; the data for this curve is shown in Fig. 2*C*).

Fig. 3 shows the effects of mutating either residue on responses to the full agonist glycine. Traces in *panel A* show that the time course of agonist responses was not affected by the mutations. There was a clear pattern of effects on the activity of glycine: shortening the residue side chain and removing its charge by replacing glutamate or arginine with alanine had only negligible effects in either the 103 or 131 positions. This was true for both the potency of glycine (Fig. 3*C*, Table 1) and for its efficacy, as both the E103A and R131A mutants maintained a high maximum *P*_open_ (0.97 ± 0.01 and 0.95 ± 0.01, *n* = 11 and 16, respectively; *top traces* in Fig. 3*B*; Table 1).

The picture was different when the side chain charge was reversed. In R131E there was a substantial decrease in the channel sensitivity to glycine (to an EC_50_ of 1.6 ± 0.2 mm, *n* = 4; Table 1) accompanied by a small decrease in maximum *P*_open_ (to 0.93 ± 0.02). A more marked effect was seen with E103R, where the increase in EC_50_ (to 1.4 ± 0.4 mm, *n* = 8; Table 1) was associated with a clear reduction in the slope of the dose-response curve (from 1.58 ± 0.07 to 0.78 ± 0.03), an effect not seen with E103K. At 200 mm glycine, a concentration that should be maximal on the dose-response curve, the single channel *P*_open_ of E103R was 0.87 ± 0.02 (*cf*. 0.987 and 0.73 for WT and E103K, respectively; Table 1).

**FIGURE 4.**
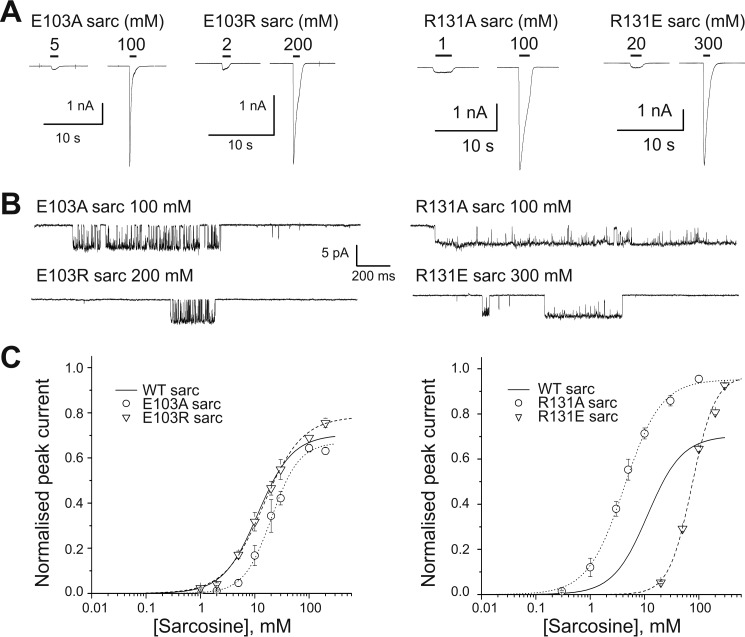
**Removing or reversing the side chain charge of Arg-131 increases the efficacy of the partial agonist sarcosine on α1 GlyR.** *A,* representative whole cell current responses to sarcosine in α1 GlyR mutants. *B,* clusters of single-channel mutant GlyR activity elicited in cell-attached patches by high concentrations of sarcosine (saturating except for the R131E mutant) *C,* sarcosine whole cell concentration-response curves for E103A, E103R, R131A, and R131E mutant GlyR (*n* = 4, 4, 5, and 6, respectively). *Curves* are scaled to the appropriate maximum *P*_open_ measured by single channel recording. The WT concentration-response curve for sarcosine is shown for comparison (*solid line*; the data for this curve is shown in Fig. 2*C*).

Fig. 4 shows that with the bulkier partial agonist sarcosine, the effect of the mutations depended mostly on their location. Mutating Glu-103 had only a small impact for both E103A and E103R. Thus we observed no shift, or a modest one, in macroscopic EC_50_ and no clear change in maximum *P*_open_ (*left panels* in Fig. 4*B*; 0.70 ± 0.03, 0.67 ± 0.08, and 0.79 ± 0.03 for WT, E103A, and E103R, respectively; *n* = 22, 8, and 31 clusters). In contrast with that, both mutations of Arg-131 markedly increased the *P*_open_ of sarcosine-evoked clusters (*right-hand panels* in Fig. 4*B*) and turned sarcosine into a nearly full agonist. For the R131A mutation, the increase in maximum *P*_open_ (0.95 ± 0.04, *n* = 37) was associated with an increase in sarcosine potency, and the EC_50_ shifted from 14 mm (WT) to 4.5 ± 0.5 mm (Table 1; *n* = 7 and 5).

For R131E, we cannot be sure that the concentration-response curve is reaching its maximum at the highest concentration of sarcosine that we could test (300 mm). Nevertheless, this concentration elicited a *P*_open_ of 0.91 (± 0.04; *n* = 21; *cf. bottom left trace* of Fig. 4*B*), higher than the WT maximum value. Equally, the EC_50_ in the mutant must be greater than 75 mm, and therefore sarcosine potency was decreased by at least 5-fold. Note that enhanced gating (proven by the increase in *P*_open_ in these mutants) *per se* should *increase* the potency of an agonist. As enhanced gating was accompanied instead by a sizeable *decrease* in potency, the R131E mutation must also decrease sarcosine affinity for the resting receptor.

##### Reversing the Side Chain Charges on Both Glu-103 and Arg-131: the Effect of the Double E103R/R131E Mutation on the Agonist Action of Glycine and Sarcosine

**FIGURE 5.**
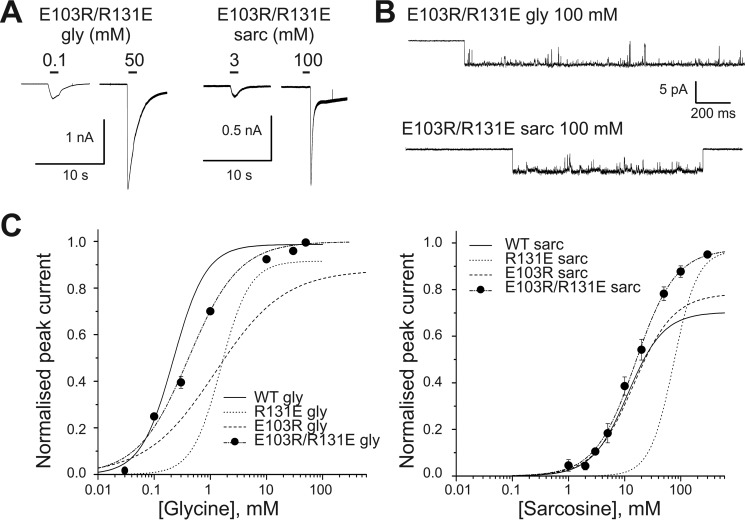
**E103R/R131E mutation rescues α1 GlyR response to glycine and sarcosine.** *A,* representative whole cell current responses to glycine or sarcosine in the double charge reversal mutant E103R/R131E GlyR α1. *B,* clusters of single-channel mutant GlyR activity elicited in cell-attached patches by saturating concentrations of glycine and sarcosine, respectively. *C,* glycine and sarcosine whole cell concentration-response curves in WT and double mutant GlyR (*n* = 5 and 8, respectively). *Curves* are scaled to the appropriate maximum *P*_open_ measured by single channel recording. The WT concentration-response curves and the single-mutation curves for both glycine and sarcosine are shown for comparison (*solid line* for WT; the data for this curve is shown in Fig. 2*C*).

Fig. 5*C* (*left panel*) shows that the double E103R/R131E mutation substantially rescued the effects on receptor function of the single charge reversal mutants. This was particularly clear in the case of glycine, with an EC_50_ in the double charge reversal mutant of 0.43 ± 0.04 mm (*black circles*, *n* = 5), close to the WT value of 0.23 mm (Fig. 5, *A* and *C*; *cf*. an EC_50_ of 1.4 ± 0.4 mm for E103R and 1.6 ± 0.2 mm for R131E; shown as *dashed* and *dotted curves* in Fig. 5*C*; Table 1). The cluster single channel trace in Fig. 5*B*, *top*, shows that the maximum *P*_open_ elicited by glycine in the double mutant is also similar to that of WT GlyR (0.996 ± 0.001 and 0.987 ± 0.002, *n* = 9 and 49, respectively).

The picture is slightly different for sarcosine. Here the double mutant has a sarcosine EC_50_ (Fig. 5, *A* and *C*) very similar to that of WT receptors (15 ± 2 and 14 ± 1 mm for the double mutant and wild type, respectively; *cf*. 17 ± 3 mm for E103R and at least 75 ± 2 mm for R131E). However, the maximum *P*_open_ elicited by sarcosine in the double mutant has remained high (0.97 ± 0.01) and resembles more the reverse charge mutant R131E (where the maximum *P*_open_ is at least 0.91 ± 0.04) than it does the WT receptor (0.70 ± 0.03). Thus, most of the effects of single charge reversal mutations of these residues are rescued by swapping their side chains, supporting the hypothesis that they form an intersubunit salt bridge.

## Discussion

Our main finding is that residues Glu-103 in loop A and Arg-131 in loop E of the canonical GlyR agonist binding site are important determinants of agonist gating efficacy, and that their influence is agonist-dependent.

### 

#### 

##### The Startle Disease Mutation E103K in Loop A of the Agonist Binding Site Acts by Impairing Glycine Gating

This mutation reduced the efficacy of the full agonist glycine on human α1 GlyR, as it decreased the maximum *P*_open_ produced by the amino acid from 0.987 to 0.73. This effect was associated with a relatively small loss in glycine potency, which brought the glycine EC_50_ from 0.23 to 0.71 mm. This loss in potency can be entirely accounted for by the reduction in glycine efficacy. The reason is as follows. The maximum *P*_open_ measured in a single channel cluster depends only on effective efficacy *E*_eff_ (defined as in Ref. 22, as *EF*/(*EF* + *F* + 1), where *E* is the opening equilibrium constant and *F* is the equilibrium constant for the pre-opening flip conformational change). Maximum *P*_open_ = *E*_eff_/(*E*_eff_ + 1). Our *P*_open_ data imply a decrease in *E*_eff_ of ∼20–25-fold (from 70 in WT receptors to 3 in the E103K GlyR). We know that the GlyR requires three molecules of agonist to open effectively (21) and we can therefore estimate that the 20-fold reduction in efficacy observed for E103K should by itself increase agonist EC_50_ by ∼3-fold (the cube root of 20) (23). This shift in EC_50_ is close to the one we observed in our experiments, and suggests that the E103K mutation does not cause major changes in the affinity of glycine for the binding site in its resting state.

This is in line with the structural information that Glu-103 does not contact the glycine molecule directly, and makes this residue particularly interesting, as it is a strong gating determinant for glycine, despite its position in the binding site. Differences in efficacy are translated into differences in gating, and reflect how much different agonists stabilize the active form of the channel. In pLGIC this is determined by the initial conformational changes and pre-open intermediate states (flip/primed/catch-and-hold) (22, 24–26). At the binding site, efficacy is reflected by the increase in microscopic affinity for the agonist as the channel enters the pre-open intermediate state. Our data suggest that E103K must reduce this affinity increase, while leaving the basal affinity (for the resting state) unchanged. A great deal more molecular dynamics simulation data with the two agonists will be required to clarify how this effect maps to structure and the binding domain changes with activation. Further probing the role of this residue with conventional mutagenesis is limited by the restricted range of replacement side chains that can be introduced. Nevertheless, we found that mutating this residue to Arg instead of Lys had a much greater effect on glycine potency, with at least a 16-fold decrease. This effect is no longer purely on gating, but must impair also resting binding affinity, perhaps because the greater bulk of the positively charged side chain of Arg *versus* Lys causes some repacking. Employing unnatural amino acid mutagenesis to produce subtler side chain changes is likely to be needed.

Thus, despite its position in the binding site, startle disease mutation E103K acts by affecting channel gating, in line with other GlyR channelopathy mutations whose mechanism has been analyzed, such as K276E, A52S, and Q266H (10–12, 27), with the exception of N46K (14). We can estimate roughly the effects of E103K on a glycinergic synaptic current by calculating the time course and open probability of the channel in response to a synaptic glycine pulse (13). We used the rate constants in the mechanism we established for homomeric α1 GlyR (28) and reduced the forward rate constants of the initial conformational changes so that the mechanism reproduces the properties of E103K we observed here. We found that the main effect of the mutation is a substantial reduction in current amplitude, with a drop in peak *P*_open_ from 0.73 to 0.13. Our calculations were done for a homomeric GlyR, and synaptic GlyR are heteromers, but it is reasonable to assume that in heteromers we would see a similar functional impairment, perhaps mitigated by the presence of the WT β subunit.

##### Mutating Residues Glu-103 and Arg-131 Has Different Effects on Glycine and Sarcosine

Our single channel recordings gave direct measurements of agonist efficacy, but we found that the pattern of effects on the two agonists of the five single mutations and the double mutation was quite complex and hard to interpret in its entirety.

For glycine, charge reversal mutations produced loss-of-function phenotypes, which were somewhat different for Glu-103 and Arg-131. E103K produced a major decrease in glycine maximum *P*_open_ which, as discussed above, was not accompanied by detectable changes in affinity. Glycine is a very efficacious agonist on GlyR, with an *E*_eff_ of 70, and this makes it easier to detect losses of efficacy than gains in efficacy. Are we likely to miss increases in glycine opening efficacy (*cf*. the ones observed for sarcosine)? Given maximum *P*_open_ = *E*_eff_/(*E*_eff_ +1), we would expect to be able to detect *decreases* in glycine efficacy greater than ∼4-fold. An increase in glycine efficacy cannot change the maximum *P*_open_, but should still be detectable, because it is expected to increase the potency of glycine, as EC_50_ is inversely proportional to *E*_eff_. The detection of such an effect could nevertheless be confounded by concurrent changes in *K_d_*.

The situation is different for sarcosine. Sarcosine is a partial agonist (*E*_eff_ about 2.5), and therefore its maximum *P*_open_ should change clearly in response to both gain and loss of function mutations. For sarcosine, the effects of mutating Glu-103 were negligible for Ala or Arg mutations (Fig. 4*C*) and substantial for the Lys mutation (Fig. 2*c*), which we could not characterize completely because achieving saturating concentrations became osmotically impossible. Somewhat surprisingly, both of the two mutations tested for Arg-131 enhanced sarcosine gating, but the two mutations had opposite effects on potency: R131A increased sarcosine potency, but R131E decreased it, implying that R131E must cause also a decrease in sarcosine binding affinity. In contrast with that, R131E had produced a small loss in both efficacy and affinity for glycine. The differences in the effects of Arg-131 mutations on glycine and sarcosine efficacy show that the determinants of efficacy are different for the two agonists.

This is the first time that such different effects of a mutation on two agonists has been documented at single channel level for GlyR and shown to involve opposite changes in the efficacy of the two agonists. Other positions in loop A of GlyRs (Lys-104, Phe-108, and Thr-112) have been mutated in oocyte-expressed human α1 GlyR (29) in an alanine-scan and found to produce gain of function, general or confined to a subset of the agonists. These mutations are also likely to affect gating, as they increased the maximum whole cell response to the partial agonist taurine (relative to glycine).

##### Is Glu-103 Forming a Salt Bridge with Arg-131?

Both our homology model and the recent structural information on GlyR are consistent with the possibility that the side chains of loop A residue Glu-103 and loop E residue Arg-131 may form a salt bridge at the back of the agonist binding site and our data on the whole support the existence of this salt bridge.

The strongest evidence in favor comes from the double mutant experiments, in which swapping the side chains of residues Glu-103 and Arg-131 rescued most of the effects of the single point mutations. The exception was the increase in sarcosine efficacy seen with Arg-131 single mutations, which persisted in the double mutant. We do not know why this is the case. In the double mutant, the salt bridge may have the right length, but the interacting charged moieties (guanidinium in Arg and carboxylate in glutamate) should be in a different position, and lie closer to the complementary side of the site. Against the salt bridge hypothesis is the finding that eliminating the charge on either residue (by Ala mutations) had practically no effect on the GlyR activation by glycine. In any mutant, it is possible that the bond that we try to disrupt is replaced by a different interaction, but we are entering the realms of speculation.

Our data show that it is not easy to understand the network of interactions at the binding site even if we have unambiguous measurements of efficacy by single channel recording and a validated homology model. Exploring the salt bridge hypothesis further will require extensive molecular dynamics investigation of the conformation of the binding pocket, and how its interactions with different agonists change as the channel activates. This will require a large amount of simulation that we have just begun and which is beyond the scope of the current paper. These investigations would then suggest additional functional experiments, which could include subtler residue changes with unnatural mutagenesis, and the formation of covalent disulfide bonds on activation.

## Experimental Procedures

### 

#### 

##### Homology Modeling

The human α1 GlyR model was constructed from the structure of *Danio rerio* α1 GlyR (glycine-bound open state; PDB code 3JAE) (17). The protein sequences for the two GlyRs (UniProt P23415 and O93430, respectively) were aligned using Muscle (30) and edited manually. 100 models were created with MODELLER version 9.16 (31) and out of the intersection of the top 10 of both DOPE score (32) and molecular PDF (31) the model with the best QMEAN score (33) was chosen for simulation. The termini of the model were capped with N-terminal acetyl and C-terminal acetamide with Maestro (Schrödinger Release 2013-2, Ref. 58) and hydrogens were added to the protein with the pdb2gmx tool of Gromacs 5.1 (34), assigning all side chains a protonation state at physiological pH (7.4).

##### Ligand Docking and Parameterization

The structures of the ligands glycine and sarcosine were taken from the ZINC ligand database (35). For each ligand and binding pocket, 50 binding modes were generated on a spherical search space of 12 Å diameter centered on the aromatic box with the docking program Gold (36) and the best-ranked one of each was selected as the final pose. The ligands were parameterized with AM1-BCC charges (37) and parameters from the general Amber force field (38) using ACPYPE (39).

##### System Set Up

Simulations were set up using a coarse-grained approach with the Martini force field version 2.2 using martinize (40) to coarse-grain the protein model. The overall systems were set up in a hexagonal prism as the periodic simulation box using insane (41), inserting the protein model in a 1-palmitoyl-2-oleoyl-*sn*-glycero-phosphocholine (POPC) bilayer, solvating in water, and adding sodium and chloride ions to neutralize the net charge and simulate a physiological concentration of 150 mm. Gromacs 5.1.x (34) was used for energy minimization and subsequent 500-ps constant temperature, constant volume (NVT)-, and 1-μs constant temperature, constant pressure (NPT) equilibration. Simulation details were based on the recommended new-rf.mdp parameters (42) and appropriately adjusted. The final frame was then converted to all-atom resolution via backward (43). The original all-atom protein model was substituted into the system with the ligands in the poses predicted by docking.

##### All Atom Molecular Dynamics (MD) Simulations

The energy minimization and dynamics simulations were performed using Gromacs 5.1.x (34) and the following force fields: AMBER99SB-ILDN (44) for protein and ions, TIP3P for water (45), Slipids (46–48) for lipids, and the general Amber force field (38) for the ligands. Periodic boundary conditions in all three spatial dimensions were applied. Each system was energy-minimized using the steepest descent algorithm until the maximum force fell below 1000 kJ mol^−1^ nm^−1^. During all phases of equilibration, position restraints were applied to the protein heavy atoms and all ligand atoms with a force constant of 1000 kJ mol^−1^ nm^−2^. 100 ps of NVT equilibration at 310 K were followed by 30-ns NPT equilibration at 1 bar. Production runs were run with no restraints for 500 ns in the NPT-ensemble. The van der Waals interactions were cut off at 1.0 nm and a dispersion correction was applied to energy and pressure. Electrostatic interactions were treated using the smooth Particle Mesh Ewald method (49, 50), where the real space contribution was cut off at 1.0 nm and the reciprocal energy term obtained in *k*-space was calculated on a grid with 0.12 nm spacing using 4th order B-splines for interpolation. The Verlet cut-off scheme was used to generate a pair-list with buffering using a tolerance of 0.005 kJ/mol/ps for pair-interactions per particle. All bonds involving hydrogens were constrained with the LINCS algorithm (51), allowing for a time step of 2 fs (only in the NVT equilibration a shorter time step of 1 fs was used). Temperature was maintained at constant physiological 310 K using the Bussi thermostat (52) with a coupling constant of 0.1 ps. In all isobaric simulations a barostat was coupled to the system in a semi-isotropic manner to maintain a pressure of 1 bar with a 1-ps coupling constant and the isothermal compressibility set to 4.5 × 10^−5^ bar^−1^. The Berendsen barostat (53) was used in the NPT equilibration runs and the Parrinello-Rahmann barostat (54) for production runs.

##### Heterologous Expression in Human Embryonic Kidney Cells

Human embryonic kidney 293 cells (HEK 293) (from American Type Culture Collection) were grown at 37 °C in a humidified 95% air, 5% CO_2_ incubator in Dulbecco's modified Eagle's medium (DMEM; Gibco) supplemented with 10% (v/v) heat-inactivated fetal bovine serum, 100 units/ml of penicillin G, 100 μg/ml of streptomycin sulfate, and 2 mm l-glutamine (all from Invitrogen). Cells were passaged after reaching 70–80% confluence, every 2–3 days, up to 30 times.

Cells were plated on poly-l-lysine-coated glass coverslips (Sigma and VWR, respectively) in 35-mm culture dishes (Scientific Laboratory Supplies, SLS) containing 2 ml of DMEM, and were then transfected by the calcium phosphate-precipitation method (55) with pcDNA3.1 plasmids, one coding for the human glycine receptor subunit α1 (GenBank^TM^ number P23415) and one coding for the enhanced green fluorescent protein (eGFP) to allow detection of transfected cells. In addition to that, empty pcDNA3.1 plasmid was added to ensure optimum expression levels. The proportion of empty plasmid varied depending on the channel expressed. For wild type channels, the final DNA mixture contained 2% GlyR α1 cDNA, 18% eGFP cDNA, and 80% empty pcDNA3.1 plasmid. The total amount of final DNA mixture was 3 μg/plate. The transfection medium was washed off and replaced by fresh DMEM 4 h after transfection. Electrophysiological experiments were performed 1–2 days after transfection.

Point mutations were introduced using the QuikChange site-directed mutagenesis kit (Stratagene). Mutant sequences were confirmed by sequencing of the full open reading frame by Source BioScience LifeSciences (Nottingham, UK).

##### Whole Cell Recordings

These were obtained at least 24 h after transfection. Patch-pipettes were pulled from thick-walled borosilicate glass (GC150F; Harvard Apparatus, Edenbridge, UK) with a Sutter P-97 pipette puller (Sutter Instruments Co.) and fire-polished to a resistance of 3–5 MΩ when filled with internal solution containing (in mm): 101.1 potassium gluconate, 11 EGTA, 1 CaCl_2_, 1 MgCl_2_, 10 HEPES, 20 tetraethylammonium chloride (TEA-Cl), 2 MgATP, 40 glucose, and 6 KCl; the pH was adjusted to 7.4 with NaOH. All solutions were filtered through a 0.2-μm Cyclopore track-etched membrane (GE Healthcare UK Ltd., Little Chalfont, UK) to remove impurities. The access resistance was below 10 mΩ and compensated by at least 70%. The bath solution contained (in mm): 20 Na gluconate, 112.7 NaCl, 2 KCl, 2 CaCl_2_, 1.2 MgCl_2_, 10 HEPES, 10 TEA-Cl, and 30 glucose; the pH was adjusted to 7.4 with NaOH. Whole cell currents were elicited by agonist application via U-tube application (56) and were recorded at a nominal holding potential of −50 mV with an AxoPatch 200B amplifier (Molecular Devices). The actual holding potential, corrected for the junction potential of 11 mV (calculated in pClamp10; Molecular Devices), was −61 mV. Recordings were prefiltered at 5 kHz with a four-pole low-pass Bessel filter, digitized at sampling rate of 20 kHz with a Digidata 1322A, and stored directly on a computer hard drive via Clampex10.2 software (all Molecular Devices).

The U-tube position was optimized before the experiment by the application of diluted bath solution (*e.g.* 50:50, bath solution:water) to an open tipped recording pipette, until the 10–90% exchange time was better than 100 ms. Average exchange time was less than 50 ms. A full concentration-response curve was obtained in each cell. To monitor the run-down/up of responses during recording, a standard concentration of agonist (usually saturating concentration) was applied every third or fourth response. Only cells in which rundown was less than 30% were accepted for further analysis and no correction for rundown was applied. The Hill equation was fitted to each individual concentration-response curve (program CVFIT; https://github.com/DCPROGS), to estimate EC_50_ and *n*_H_ values. For the purpose of display only, responses were then normalized to the fitted maximum in each cell. These normalized responses were pooled and refitted with the Hill equation for the display.

##### Single-channel Recordings

These were obtained in the cell-attached configuration at least 24 h after transfection. Extracellular solution was made with HPLC-grade water, filtered through a 0.2-μm Cyclopore track-etched membrane (GE Healthcare UK Ltd., Little Chalfont, UK) and contained (mm): 102.7 sodium gluconate, 4.7 KCl, 2 CaCl_2_, 1.2 MgCl_2_, 10 HEPES, 14 glucose, 15 sucrose, 20 TEA-Cl (from Sigma); the pH was adjusted to 7.4 with NaOH; osmolarity at 320 mOsm. Sylgard-coated thick-walled borosilicate pipettes fire-polished to a resistance of 10–15 MΩ were filled with extracellular solution containing a saturating concentration of agonist. Patches were voltage-clamped at a pipette potential of +100 mV with an Axopatch 200B amplifier. Data were pre-filtered at 10 kHz with the amplifier four-pole Bessel filter, digitized at 100 kHz with a Digidata 1322A and Clampex 10.2 (Molecular Devices) directly on the computer hard drive. Data were digitally filtered (low-pass Gaussian filter) to a final cut-off frequency of 4–6 kHz. At high agonist concentrations openings occurred in clusters delimited by long closed (desensitized) intervals (see Fig. 2*B*). Clusters are likely to originate from the activity of a single ion channel molecule and were used for *P*_open_ measurements (57), selecting clusters longer than 100 ms and with more than 10 openings. First, channel activity in the selected clusters was idealized by half-amplitude threshold method (Clampfit 10.2, Molecular Devices). Open probability was calculated as the ratio of cluster open time over total cluster length. The single-channel current amplitude was calculated as a difference between the full open level (at the beginning of each cluster; 0.1 to 10 ms stretches) and the baseline (just before each cluster; 10 ms stretches).

The open probability response to a glycine concentration pulse that approximates synaptic conditions was calculated assuming an instantaneous rise to 3 mm glycine, followed by an exponential decay with a time constant of 2.5 ms (13). Rate constants used in the calculation are those obtained by global mechanism fit to rat α1 GlyR (28). The effect of the E103K mutation was approximated by reducing by 100-fold each of the three forward rate constants for flipping (δ1, δ2, and δ3). The resulting GlyR would have a maximum *P*_open_ of 0.75 and an EC_50_ of 1.15 mm (*cf*. 0.73 and 0.71 mm measured for the E103K GlyR). Calculations were done using SCALCS. A Jupyter notebook with calculations is deposited in the SCALCS Git repository (https://github.com/DCPROGS/SCALCS/blob/master/notebooks/Calculate_Popen_SynapticCurrent_alpha1GlyR_Flip.ipynb). All electrophysiological recordings were carried out at a temperature of 19–21 °C.

Sarcosine was tested at 100 mm for glycine contamination by an HPLC assay: samples were resuspended in 10 μl of 50% EtOH and reacted for 30 min with the following reagent: 90% EtOH:triethylamine:phenylisothiocyanate (7:2:1). They were then evaporated to dryness at room temperature and redissolved in 100 μl of 5% acetonitrile in 0.1 m ammonium acetate. A 150 × 4.6-mm Hypersil C18 column was used. Molecules of interest were detected at 254 nm. Sarcosine (Sigma) was found to be contaminated by about 10 μm glycine per 100 mm. Sarcosine (approximately 7 g) was therefore purified by re-crystallizing it three times with 95% ethanol obtaining 2.37 g of purified compound, whose ^1^H NMR and elemental analysis were consistent with those expected for sarcosine. The glycine contamination of a 100 mm solution of purified sarcosine was below the detection limit of the HPLC assay (about 1 μm).

## Author Contributions

F. S., E. H., M. E., T. G., and R. L. conducted patch clamp experiments; F. S. and R. L. analyzed experiments; R. L. made the patch clamp figures; M. W. I., G. F., and D. J. purified sarcosine; R. Y., M. A. D., and P. C. B. conducted molecular dynamics simulations and made Fig. 1; L. G. S., R. L., and P. C. B. designed experiments; L. G. S. wrote most of the paper with P. C. B. All authors reviewed the results and approved the final version of the manuscript.

## Footnotes

5 The abbreviations used are:

pLGIC: pentameric ligand-gated ion channel superfamily
GlyR: glycine receptor
*P*_open_: open probability
TEA: tetraethylammonium
PDB: Protein Data Bank.
